# Effects of single aortic clamping versus partial aortic clamping techniques on post-operative stroke during coronary artery bypass surgery

**DOI:** 10.5830/CVJA-2013-038

**Published:** 2013-08

**Authors:** Ihsan Sami Uyar, Mehmet Besir Akpinar, Veysel Sahin, Feyzi Abacilar, Volkan Yurtman, Faik Fevzi Okur, Ugur Ozdemir, Mehmet Ates

**Affiliations:** Department of Cardiovascular Surgery, Medical Faculty, Sifa University Izmir, Turkey; Department of Cardiovascular Surgery, Medical Faculty, Sifa University Izmir, Turkey; Department of Cardiovascular Surgery, Medical Faculty, Sifa University Izmir, Turkey; Department of Cardiovascular Surgery, Medical Faculty, Sifa University Izmir, Turkey; Department of Cardiovascular Surgery, Medical Faculty, Sifa University Izmir, Turkey; Department of Cardiovascular Surgery, Medical Faculty, Sifa University Izmir, Turkey; Department of Cardiovascular Surgery, Medical Faculty, Sifa University Izmir, Turkey; Department of Cardiovascular Surgery, Medical Faculty, Sifa University Izmir, Turkey

**Keywords:** coronary bypass, stroke, partial clamp

## Abstract

**Background:**

The aim of this study was to compare the effects of single-clamping and partial-clamping techniques on postoperative stroke during coronary artery bypass surgery.

**Methods:**

Between December 2008 and December 2012, 2 000 patients who underwent coronary artery bypass grafting in two hospitals were analysed. Post-operative neurological complications were analysed retrospectively in these patients. The cases were divided into two groups: in group 1, 1 500 patients were analysed, in whom proximal anastomosis was performed with partial clamping in a beating heart (*n* = 1 500, 846 male, 654 female; mean age 63.25 ± 5.72 years; range 43–78 years). In group 2, 500 patients were analysed, in whom proximal anastomosis had been performed by other surgical teams in another hospital, with cross clamping in a resting heart with cardioplegia (*n* = 500, 296 male, 214 female; mean age 64.83 ± 8.12 years; range 41–81 years). During 30 days post-operatively, neurological deficits, stroke incidence and the relationship of the clinical situation to mortality were analysed.

**Results:**

For both groups, patients were similar in terms of patient characteristics. In group 2, cross-clamp duration and perfusion time were longer; however, time of hospital stay was similar in the two groups. Post-operative stroke was seen in 26 patients in group 1 (1.73%) and in nine in group 2 (1.8%). The difference between the two groups was not statistically significant (*p* = 0.92). All stroke patients were over the age of 55 years. Seven of the stroke patients died (21.1%). In total, 31 patients died because of multiple organ failure in the postoperative 30 days (group 1: 1.6%; group 2: 1.4%) (*p* = 0.91). Smoking, diabetes mellitus, hypertension, atrial fibrillation, peripheral vascular disease and hypercholesterolaemia were found to be factors that affected stroke development. Mean duration of hospital stay was 5.1 ± 2.8 days in group 1 and 4.9 ± 3.6 days in group 2 and the difference between the two groups was not statistically significant (*p* = 0.46).

**Conclusion:**

In patients without plaques in the aorta, performing partial clamping did not increase stroke incidence.

## Abstract

Neurological complications are undoubtedly among the most important adverse outcomes of coronary artery bypass surgery. Neurological problems increase morbidity and mortality considerably. Although techniques performed during cardiac surgery have progressed, neurological complications have not disappeared completely. The real causes of these complications may be problems associated with atherosclerosis that is present before the operation or with surgical technical failure. Advanced age, carotid artery disease and severe calcification of the aorta are the main factors that increase the risk of neurological complications.^1^-^3^

There are many studies in the literature on the prevention of these problems, and they suggest technical solutions. However, concerns about the safety of these techniques and the neurological complications still exist.

Patients with similar demographic characteristics who were treated with coronary artery bypass grafting (CABG) at our clinic were evaluated retrospectively in this study. The results obtained by a team using a single aortic clamp for the proximal anastomosis were compared with those obtained by a team utilising partial clamping for the proximal anastomosis. The study sought to observe whether either of these techniques increased the incidence of post-operative stroke.

## Methods

In this study, 2 000 patients who had undergone CABG in two hospitals between December 2008 and December 2012 were evaluated retrospectively for neurological complications and mortality in the post-operative period. The cases were divided into two groups. Data from the 1 500 patients in whom proximal anastomosis had been performed with partial clamping were evaluated as group 1 (*n* = 1 500; 846 males, 654 females; age 63.25 ± 5.72 years; range of 43–78 years). The data from the patients (treated by another surgical team in another hospital) in whom proximal anastomosis had been performed in the stopped heart with cross clamping were evaluated as group 2 (*n* = 500; 296 males, 214 females; mean age 64.83 ± 8.12 years; range 41–81 years). The data for these two groups were compared.

The study was planned, and approval of the hospital ethics committee was obtained. The informed consent forms of all patients were seen in chart review, and cases that did not have an informed consent form were excluded. Patients with plaques in the aorta and with a history of corticosteroid, salicylate, dipyridamole or anticoagulant use, those that had a coagulation or platelet dysfunction, and cases having simultaneous valve surgery, aortic surgery, ventricular aneurism resection or carotid endarterectomy were also excluded from this study. Cases in whom stenosis of the carotid artery was considered critical were excluded. Patients who had undergone re-operation were also excluded.

Patients older than 60 years of age, those with a history of stroke or transient ischaemic attack before the operation, and cases in whom a systolic murmur was detected over the carotid artery were all routinely examined by carotid Doppler ultrasonography.

The development of neurological deficits and strokes in the 30-day post-operative period, and the association of this clinical situation with mortality was assessed. These cases were followed up in co-operation with a neurologist. Cranial magnetic resonance imaging and computerised tomography were used in the diagnosis.

Midalozam 5 mg was administered intramuscularly to all patients one hour before the intervention as pre-medication. Cefazolin 1 g was administered intravenously before induction of anaesthesia and continued in repeated doses (twice a day) until chest tube removal. In case of infection, a swab was taken from the wound and following the outcome of cultures, targeted antibiotics were administered.

Internal thoracic artery (ITA) and saphenous vein grafts were prepared after median sternotomy. Cardiopulmonary bypass was started with arterial cannulation of the ascending aorta and two-stage venous cannulation from the right atrial auricle. Non-pulsatile extra-corporeal circulation (ECC) (cardiopulmonary bypass) was started with a Sarns roller pump (Sarns, Fort Myers, FL,USA) at 2.4–2.6 l/m^2^/min, and mild–moderate hypothermia (oesophageal temperature 26–28°C) was obtained. A membrane oxygenator (Dideco, Mirandola, Italy) was used for oxygenation during ECC.

The haematocrit values were kept between 20 and 25%, and mean perfusion pressure measured from the radial artery was kept between 50 and 80 mmHg during bypass. Multi-dose antegrade blood cardioplegia was used in both groups.

Before placement of the cross clamping, the arterial pressure was lowered to 30 mmHg for a few seconds in a controlled fashion with ECC support, and the aorta was evaluated manually in detail. Partial clamping was not used in patients in whom the presence of plaques in the aorta was suspected. For patients in whom no atherosclerotic plaques were found in the aorta, it was clamped at the aortic root.

Cardiac arrest was obtained by applying 1 500 ml isothermic blood cardioplegic solution antegradely from the aortic root, and topical cold saline was applied in all patients. Blood cardioplegic solution doses for maintenance were administered consequently. Cardioplegic solution at 37ºC, with the heater circulatory system in the cardioplegia set (Dideco, Mirandola, Italy), was administered to obtain a controlled reperfusion before the cross clamp was removed in both groups.

All of the proximal anastomoses were done with an aortic partial clamp in group 1. The clamp was removed when the last proximal anastomosis was completed, before the suture material was tied; bleeding of the aorta for at least 10 seconds was permitted before tying.

In group 2, each patient was warmed while the proximal anastomoses were performed. The proximal anastomoses were done under cross clamping in the resting heart. The sutures were not tied after the last proximal anastomosis, and bleeding of the aorta was permitted for at least 10 seconds in this region after the clamp was removed. The patients were kept under observation in the intensive care unit for at least six hours with mechanical respiratory support.

## Statistical analysis

The results are presented as mean ± standard deviation. The data were evaluated with multivariate logistic analysis, Student’s *t*-test and chi-square test. In all studies, *p*-values < 0.05 were considered statistically significant.

## Results

There were no statistically significant differences between the two groups in terms of mean age, gender, morbid obesity, smoking habit, hypertension, diabetes mellitus, and chronic pulmonary or renal disease (*p* > 0.05). There were also no significant differences in terms of functional capacity according to New York Heart Association (NYHA) classification, blood cholesterol level, family history, accompanying peripheral artery disease and history of cerebrovascular disease (*p* > 0.05). The mean ejection fraction (EF) values of the patients at pre-operative echocardiographic evaluation were 45.4 ± 5.23 in group 1 and 46.4 ± 2.31 in group 2, and the difference was not statistically significant (*p* = 0.067). Comparisons of the demographic data of the patients are summarised in Table 1.

**Table 1:** Demographic, Clinical And Procedural Data For Study Patients

	*Group 1 (n = 1500)*		*Group 2 (n = 500)*		
*Parameters*	*Patient number*	*%*	*Patient number*	*%*	p*-value*
Age (mean ± SD)	63.25 ± 5.72		64.83 ± 8.12		0.079
Female gender	654	43.6	214	42.8	0.46
Cardiac data					
Acute MI	93	6.2	29	5.8	0.065
Heart failure (pre-operative)	486	32.4	144	28.8	0.058
Risk factors					
Smoking	564	37.6	191	38.2	0.95
Hypertension	768	51.2	254	50.8	0.73
Morbid obesity	276	18.4	96	19.2	0.66
Dyslipidaemia	1023	68.2	339	67.8	0.97
Family history	357	23.8	109	21.8	0.39
Peripheral vascular disease	192	12.8	56	11.2	0.38
MI (pre-operative)	648	43.2	214	42.8	0.73
DM	675	45.0	223	44.6	0.73
COLD	333	22.2	109	21.8	0.90
CRF	84	5.6	26	5.2	0.13
NYHA class (mean ± SD)	3.24 ± 7.1		3.18 ± 1.2		0.65
EF (mean ± SD) (pre-operative)	45.46 ± 5.23		46.41 ± 2.31		0.067
LMCA lesion	375	31.51	685	29.65	0.567

Mean ± SD, mean ± standard deviation; MI, myocardial infarction; DM, diabetes mellitus; COLD, chronic obstructive lung disease; CRF, chronic renal failure; NYHA, New York Heart Association; EF, ejection fraction; LMCA, left main coronary artery.

The operative data of the patients, such as time of cross clamp, time of perfusion, mean number of distal anastomoses, inotropic agent support, amount of post-operative drainage, intra-aortic balloon use, peri-operative myocardial infarction, duration of intubation, duration of stay in the intensive care unit (ICU), and post-operative complications were compared. The results of these comparisons are summarised in Table 2.

**Table 2:** Complications And Mortality After Coronary Artery Bypass Grafting

	*Group 1 (n = 1500)*	*Group 2 (n = 500)*	p*-value*
Number of distal anastomoses (mean ± SD)	2.98 ± 1.9 (1–5)	3.12 ± 2.1 (1–4)	0.96
Time of cross clamp (min) (mean ± SD)	41 ± 4.3	69.6 ± 1.3	0.001
Time of perfusion (min) (mean ± SD)	67.3 ± 3.6	76.7 ± 2.2	0.001
Positive inotropic support (%)	31.2	29.4	0.43
IABP (%)	1.26	0.8	0.001
Time in operating room (min) (mean ± SD)	187.95 ± 2.32	185.25 ± 7.35	0.072
Using grafts			
LITA, *n* (%) (for LAD coronary artery)	2275 (98.48)	483 (96.62%)	0.001
Blood transfusion (units)	3.1 ± 1.4	2.8 ± 2.7	0.001
Days in ICU (mean ± SD)	1.2 ± 2.1	1.8 ± 2.3	0.001
Total days in hospital (mean ± SD)	5.1 ± 2.8	4.9 ± 3.6	0.46
Post-operative bleeding (ml)	550 ± 2.6	490 ± 2.6	0.001
Intubation time (hour) (mean ± SD)	7.86 ± 9.2	12.67 ± 4.8	0.001
Re-operation for bleeding (%)	0.6	0.4	0.86
Peri-operative MI (%)	1.9	2.1	0.92
Stroke, *n* (%)	26, 1.73	9, 1.8	0.92
30-day mortality, *n* (%)	24, 1.6	7, 1.4	0.91

Mean ± SD, mean ± standard deviation; IABP, intra-aortic balloon counter pulsation; LITA, left internal thoracic artery; LAD, left anterior coronary artery; ICU, intensive care unit; CVE, cerebrovascular events; AF, atrial fibrillation.

Post-operative stroke was seen in 26 patients in group 1 (1.73%), and in nine patients in group 2 (1.8%). The difference was not statistically significant (*p* = 0.92). All patients who suffered from stroke were older than 55 years. In a detailed analysis of the group of patients with stroke, the frequency of smoking was 24%, diabetes 67%, hypertension 72%, atrial fibrillation 35%, peripheral arterial disease 74%, and hypercholesterolaemia 68%. Echocardiographic left ventricular ejection fraction (LVEF) was lower than 45% in all patients (Table 3).

**Table 3:** Risk Factors For Stroke After CABG In Multivariate Logistic Analysis

*Parameters*	*Stroke patients (n = 35)*	*Populatıon (n = 2000)*	p*-value*
Age (mean ± SD)	65.26 ± 21.4	63.85 ± 5.72	0.14
LMCA disease (%)	17.9	18.4	0.85
IABP use (%)	1.23	1.15	0.925
Time of perfusion (min) (mean ± SD)	79.65 ± 48.12	71.15 ± 24.12	0.001
Time of cross clamp (min) (mean ± SD)	48.75 ± 29.12	36.14 ± 65	0.001
Post-operative hypotension (%)	79.8	12.4	0.001
Peripheral vascular disease (%)	74	16.5	0.001
Hypertension (%)	72	44.6	0.001
Smoking (%)	24	56.7	0.001
AF (%)	35	22.6	0.001
Dyslipidaemia (%)	68	94.5	0.001
DM (%)	67	86.7	0.001
CRF (%)	3.9	4.3	0.792
LVEF (mean ± SD)	38.27 ± 26.31	46.12 ± 12.31	0.001

Mean ± SD, mean ± standard deviation; LMCA, left main coronary artery; IABP, intra-aortic balloon counter pulsation; AF, atrial fibrillation; DM, diabetes mellitus; CRF, chronic renal failure; LVEF, left ventricular ejection fraction.

A total of 31 patients [24 patients from group 1 (1.6%) and seven patients from group 2 (1.4%)] were lost during the 30-day post-operative period due to multiple organ failure that had developed after low cardiac output. The difference between the groups was not significant (*p* = 0.91). The 30-day mortality rate in all patients included in this study was 1.55% (31 patients). The mean duration of stay in hospital was 5.1 ± 2.8 days in group 1 and 4.9 ± 3.6 days in group 2, and the difference was not significant (*p* = 0.46).

## Discussion

Major neurological problems are among the most feared complications after CABG with cardio-pulmonary bypass. Neurological complications have a major effect on post-operative morbidity and mortality and the outcome may be catastrophic for both the patient and surgeon. The rate of stroke after cardiac surgery is reported to be between 1 and 5% in published studies and this may increase to 9% in patients over 75 years of age.^1^,^4^ Indeed, mortality may reach 28% in the latter cases.^5^-^7^

Cerebral damage and neurological complications due to this damage have many causes, including embolisations originating in the heart and aorta (air, particles from ruptured plaques, fatty particles), cerebral hypo-perfusion, bleeding, carotid artery disease, and metabolic causes such as toxic mediators and cytokines released during prolonged ECC.^8^,^9^ The dimensions of the neurological damage are dependent on the extent of embolisation and the affected region.

There are publications in which single aortic clamping is recommended for proximal and distal anastomosis in CABG surgery, in order to decrease the rate of such complications.^4^,^8^,^10^,^11^ Aranki *et al.* 12 reported a decrease in both hospital mortality and cerebral damage with the use of the single-clamp technique. Marshall *et al.*^13^ have shown that most of the embolisations occurred during manipulation of the aorta, especially when the clamp was removed from the aorta.

In the Framingham study, the rate of stroke was 3.5 times higher in patients over 65 years, with a calcification in their aorta. The rate of sudden death due to coronary artery disease was found to be twice as high in patients younger than 65 years, with radiological aortic calcifications, in comparison with patients without aortic calcifications in the same study.^14^,^15^

Akpınar *et al.*^5^ reported performing coronary artery bypass grafting with only arterial grafts, by inducing ventricular fibrillation with deep hypothermia in 23 patients who had advanced calcifications in the aorta. The mean age of these patients was 65 years, and major neurological complications were not observed in any of them. We utilised this method in only one of our patients in this series, a patient on whom we had operated in the past five years. However, like other patients in whom plaques were detected in the aorta, this patient was not included in this study.

In a study by Orhan *et al.*,^1^ partial clamping was compared with single clamping during coronary bypass operations, and no statistical differences were observed between these groups in terms of stroke and neurological problems. In a study by Us *et al.*,^8^ neurological complications were not observed in patients in whom the single-clamp technique was used, whereas neurological complications were statistically significantly higher in those in whom partial clamping was used. Güden *et al.*^16^ recommend the single-clamp technique in CABG surgery, as this approach minimises possible embolisations from the aorta and neurological complications, and decreases the duration of ischaemia.

In the present study, we were not able to show that the single-clamping technique was superior to the partial-clamping technique during coronary artery bypass operations, in terms of frequency of neurological damage. We found that the mortality rate was the same in both groups.

The single-clamp technique may not be sufficient to minimise neurological complications by itself. Sequential anastomosis may be preferred in order to decrease the number of proximal anastomoses in patients with plaques in the aorta. As the sequential-anastomosis technique is not used routinely in our clinic, this was not included among the parameters evaluated in the present study. Also, replacement of the ascending aorta under hypothermic circulatory arrest, which was recommended by Kouchokos *et al.*^17^ for patients with severely calcific aortas, may be considered as an alternative method. The authors reported a mortality rate of 4.3%, without neurological complications.

Revascularisation with the ‘no-touch’ technique or ‘off-pump’ coronary bypass grafting, which were described by Mills *et al.*,^18^ may be applied to patients, especially those who have atherosclerotic plaques in their ascending aortas. Coronary bypass surgery with the beating heart technique may be an alternative method, if proximal anastomoses are used for a region outside the ascending aorta. Intra-operative ultrasonographic evaluation is recommended to detect the presence of plaques in the ascending aorta.^13^ This may be the best method for the prevention of neurological complications due to possible embolisations from the aorta. However, in our hospital, we could not use this method because of practical problems in the operating rooms.

Advanced age, carotid artery disease, aortic atherosclerosis, previous cerebrovascular disease, prolonged cardiopulmonary bypass, and peri-operative hypotension are reported as risk factors for stroke development after CABG.^2^,^19^ We detected prolonged cross-clamp duration, diabetes, hypercholesterolaemia, left ventricular dysfunction (EF < 40%), atrial fibrillation, peripheral arterial disease, and peri-operative hypotension as risk factors for stroke (Table 3). Partial clamping used during proximal anastomosis was not found to be a risk factor. Our observation of our patients was that the neurological damages seen after coronary artery bypass surgery were due to multiple factors; they cannot be decreased solely by means of the single-clamp technique.

Neurological problems may also occur during placement and replacement of total aortic cross clamp, aortic cannulation or aortic ‘punch’ application, as well as partial clamp use. Also, there are reports of embolidation even during manual examination of the aorta.^16^,^18^,^20^ It is known that thrombus material that was already present in the left atrium or ventricle may also cause embolism. Each of these factors may cause neurological problems.

At our clinic, we routinely created controlled hypotension for a few seconds with ECC support before replacement of an aortic clamp in patients undergoing CABG. During those few seconds, the surgeon evaluated the aorta carefully. Different techniques other than the routine could be used in patients in whom the presence of aortic plaques was detected. However, patients with aortic plaques were not included in the current study. It is probably for this reason that we found non-superiority of the single-clamping technique in comparison with partial clamping, in terms of stroke development.

Grocott *et al.*^21^ found that the S100β protein was a good marker for showing cerebral damage and that the level of this protein was highest during aortic cannulation in coronary bypass surgery. These authors also detected that the serum levels of the S100β protein were lower during aortic cross-clamp placement or replacement. It was stressed in this study that cannulation was more important in terms of post-operative stroke risk than clamp placement on the aorta.

We believe that metabolic investigations such as protein C, protein S, and anti-thrombin deficiency should be done, as well as determining S100β protein levels for post-CABG stroke and cerebral damage. We could not investigate these parameters, given that the present study was retrospective. Also, the lack of echocardiographic examination of the aorta and the lack of a prospective design were limiting factors of the present study.

## Conclusion

We believe that a surgical team may utilise either of the two techniques studied here, after the surgeon has evaluated the aorta at low aortic pressure and concluded that plaques are not present. We feel that both methods may be used safely in routine coronary artery bypass grafting.
